# Application of Metal Nanoparticle–Hydrogel Composites in Tissue Regeneration

**DOI:** 10.3390/bioengineering6010017

**Published:** 2019-02-11

**Authors:** Hui-Li Tan, Sin-Yeang Teow, Janarthanan Pushpamalar

**Affiliations:** 1 School of Science, Monash University Malaysia, Jalan Lagoon Selatan, Bandar Sunway, Subang Jaya 47500, Selangor Darul Ehsan, Malaysia; hui.tan@monash.edu; 2 Department of Medical Sciences, School of Healthcare and Medical Sciences, Sunway University, Jalan Universiti, Bandar Sunway 47500, Selangor Darul Ehsan, Malaysia; 3 Monash-Industry Palm Oil Education and Research Platform (MIPO), Monash University Malaysia, Jalan Lagoon Selatan, Bandar Sunway 47500, Selangor Darul Ehsan, Malaysia

**Keywords:** silver nanoparticle, gold nanoparticle, hydrogel, nanocomposite, tissue engineering, regenerative medicine

## Abstract

Challenges in organ transplantation such as high organ demand and biocompatibility issues have led scientists in the field of tissue engineering and regenerative medicine to work on the use of scaffolds as an alternative to transplantation. Among different types of scaffolds, polymeric hydrogel scaffolds have received considerable attention because of their biocompatibility and structural similarity to native tissues. However, hydrogel scaffolds have several limitations, such as weak mechanical property and a lack of bioactive property. On the other hand, noble metal particles, particularly gold (Au) and silver (Ag) nanoparticles (NPs), can be incorporated into the hydrogel matrix to form NP–hydrogel composite scaffolds with enhanced physical and biological properties. This review aims to highlight the potential of these hybrid materials in tissue engineering applications. Additionally, the main approaches that have been used for the synthesis of NP–hydrogel composites and the possible limitations and challenges associated with the application of these materials are discussed.

## 1. Introduction

The application of hydrogel incorporated with metal nanoparticles (NPs) has become a new emerging research area in tissue engineering and regenerative medicine. Disease, injury, and trauma often resulted in tissue damage and degeneration. For repair, replacement, or regeneration, the treatment normally involves the transplantation of tissue from the same patient (autograft) or another individual (allograft). However, the treatments are risky, because autografts can lead to donor-site morbidity due to infection and hematoma, whereas allografts might be rejected by the host immune system [1]. Furthermore, there is a huge gap between the supply and demand for organs. As of December 2018, there are about 110,000 patients waiting for lifesaving organ transplants in the United States, while there are only about 16,000 donors available in 2018 [2]. 

In order to overcome the challenges of high organ demand and biocompatibility issues, scientists in the field of tissue engineering and regenerative medicine are working on the use of scaffolds as an alternative to transplantation. These scaffolds are developed to mimic the extracellular matrix (ECM), act as structural support, and define the potential space for new tissue development as well as enhance the cell attachment, proliferation, and differentiation [3]. In addition, scaffolds can be used as the delivery vehicles of essential growth factors to manipulate and promote tissue growth [4]. 

There are various kinds of materials that have been used to facilitate and develop the tissue engineering scaffolds. The examples are metals, natural and synthetic polymers, and ceramics [5]. Among different types of scaffolds, polymeric hydrogel scaffolds have gained remarkable interest because they are biocompatible, and the structures are similar to the macromolecular-based components in the body [6]. However, the traditional hydrogel scaffolds often have poor mechanical strength and a lack of bioactive property, which limited their applications in tissue regeneration [7]. Therefore, recent studies have been working on the development of modified hydrogel via basic to advanced material-based approaches to enhance the physical and chemical properties of the scaffolds [8]. One example of the approaches is to integrate noble metal NPs such as gold (Au) and silver (Ag) NPs into the system, forming a hybrid material known as NP–hydrogel composite [9]. While improving the physical and chemical properties of the hydrogel, most of the metal NPs are bioactive and naturally possess anti-bacterial [10], anti-viral [11], and anti-inflammatory [12] actions. This provides additional advantages to the composite for tissue regeneration. 

Although noble metal NPs and hydrogel alone have been well-characterized, the research on the application of noble metal NP–hydrogel composites as tissue engineering scaffolds is still limited. In this review, we will focus on recent studies on the development of noble metal NP–hydrogel composites for tissue engineering purposes. We will also discuss the main approaches for the fabrication of the composite materials. In addition, we will point out some possible limitations and challenges related to the application of these hybrid materials.

## 2. Noble Metal NPs, Hydrogel, and NP–Hydrogel Composite

Over the years, noble metal NPs and hydrogels have been widely studied as potential biomaterials for tissue regeneration. Today, scientific innovations have led to the emergence of the composite material made of noble metal NPs and hydrogel, forming an inorganic–organic framework with improved properties. 

### 2.1. Noble Metal NPs

In the past two decades, nanoparticles have been widely studied for a wide range of applications, and noble metal nanoparticles are the attractive nanomaterials due to their uniqueness such as resistance to corrosion and oxidation, and non-reactiveness [13]. Among the noble metal NPs, Au and Ag NPs are the most commonly studied nanomaterials. The interesting properties of these noble metal NPs are their high surface-to-volume ratio, wide optical properties, ease of synthesis, and facile surface chemistry and functionalisation. These have led them to be applied in various biomedical applications such as diagnostic assays, thermal ablation, radiotherapy enhancement, and drug and gene delivery [14]. Based on the extensive in vitro and in vivo studies on different types of cancers, Au and Ag NPs are recognized as promising anti-cancer agents due to their effectiveness against drug-resistant tumor cells through distinct mechanisms [15]. In addition, Au and Ag NPs have been explored for their antimicrobial activity. Ag NPs have antimicrobial activity against a broad range of bacteria strains, including an antibiotic-resistant strain. The mechanism of action is based on the inhibition of bacterial enzymatic activities, the attenuation of DNA replication, and the disruption of bacterial cell membranes [16]. Au NPs have gained increased attention for their antimicrobial activity, but the mechanism has not yet been fully understood [16]. In recent years, Au and Ag NPs have found potential applications in tissue regeneration. The NPs were proposed to be advantageous in tissue engineering due to their small size, which facilitates their transport across the cell membranes. Furthermore, the size and surface characteristics are customizable according to desired purposes [17].

Au NPs are also known as colloidal gold. They can be easily prepared in the diameter range of three to 200 nm, as well as in different shapes such as gold nanocubes [18], gold nanostars [19], and gold nanorods [20]. The common shape is the quasi-spherical shape, because the surface energy favors the formation of spherical particles [21]. It has been found that Au NPs are potential osteogenic agents for bone regeneration [22]. It was reported that Au NPs could stimulate the differentiation of primary osteoblasts and mesenchymal stem cells through the activation of extracellular signal-regulated kinases (ERK)/mitogen-activated protein kinases (MAPK), and p38 MAPK pathways, respectively [23]. Besides, functionalized Au NPs such as chitosan-conjugated Au NPs and gellan gum-coated Au nanorods were also being tested on human adipose-derived mesenchymal stem cells and osteoblast-like cells, respectively [24,25]. It has been suggested that the behavior of stem cells is influenced by the surface functionalization of Au NPs [26]. 

For Ag NPs, they are typically one to 100 nm in diameter [27]. Similarly, Ag NPs were reported to promote the proliferation of human mesenchymal stem cells. It was proposed that the effect was related to the hypoxia-inducible factor 1-alpha (HIF-1α)-mediated upregulation of interleukin-8 (IL-8) expression [28]. In addition, the osteogenic differentiation of urine-derived stem cells induced by the treatment of Ag NPs has also been demonstrated [29]. 

Other than Ag and Au, the applications of platinum (Pt) NPs have also been explored. For example, iron-Pt (FePt) magnetic NPs were synthesized and utilized to enhance the cell infiltration and distribution of cells within the poly(lactic-co-glycolic acid) salt-leached scaffolds, with a neodymium magnet placed at the bottom [30]. The effect of Pt NPs alone has also been reported in the study conducted by Eid et al. Pt NPs were loaded into calcium phosphate scaffold for bone allograft. There was enhanced cell proliferation and attachment [31]. Nevertheless, the number of studies on the applications of Pt NPs in tissue engineering is highly limited compared to Au and Ag NPs. 

### 2.2. Hydrogel 

A hydrogel is a three-dimensional (3D) network that is composed of cross-linked synthetic or natural polymers. The common natural polymers that are being used are alginate, chitosan, collagen, and gelatin, and examples of synthetic polymers are polyethylene glycol (PEG), polyacrylamide, and polydimethylsiloxane [32]. Hydrogel possesses attractive properties such as a soft porous structure, high water content, and biocompatibility, and it tends to absorb physiological fluids [33]. Besides, the porous structure allows high permeability for oxygen, nutrients, and other water-soluble metabolites. Based on the similarities of the physical, chemical, and biological properties to those of native body tissues, hydrogel has become a useful scaffold material in tissue engineering applications [34]. 

In the study reported by Zhao et al., a photocrosslinkable gelatin hydrogel was synthesized for skin tissue engineering [35]. The mechanical and degradation properties of the hydrogel were tunable by varying the concentration of gelatin methacrylamide prepolymer solutions. The hydrogels of all the polymer concentrations were shown to enhance the growth and differentiation, eventually supporting the formation of the stratified epidermis [35]. Besides, the hydrogel scaffold is also a potential candidate for cartilage tissue engineering. Wang et al. fabricated an injectable hydrogel that consisted of a four-arm star PEG functionalized with vinyl sulfone and a short dithiol crosslinker. Murine chondrocytes were encapsulated in the hydrogel and transplanted into severe combined immunodeficiency (SCID) mice. The chondrocytes within the hydrogel matrix proliferated and maintained their phenotype [36]. For the injuries in the central nervous system, the tissue repair is often challenging due to the lack of an ECM and vascularization, which prevents the infiltration of cellular elements and axon regeneration. In an in vivo study, the injection of imidazole-poly(organophosphazenes) hydrogel has induced the remodeling of ECM and stimulated the tissue repair after central nervous system injuries [37]. Besides, hydrogels have also been studied for many other tissues such as cardiac, kidney, and liver tissue regeneration [38,39,40]. 

### 2.3. NP–Hydrogel Composite 

Recently, studies in different areas have shown that the addition of NPs has widened the applications of the hydrogel in catalysis, electronics, biosensing, drug delivery, nanomedicine, and environmental remediation due to the property enhancement [41]. The examples of NPs are polymeric NPs (polymer NPs, dendrimers, and hyperbranched polyesters), inorganic/ceramic NPs (hydroxyapatite, silica, silicates, and calcium phosphate), and metal/metal-oxide NPs (Au, Ag, and iron oxide) [42]. In one of the studies, magnetic Fe_3_O_4_ NPs have been loaded into chitosan/PEG hydrogel. The incorporation of the magnetic NPs has resulted in the higher viability and osteogenic differentiation ability of mesenchymal stem cells [43]. The incorporation of hydroxyapatite in silk fibroin hydrogel has promoted the osteogenic differentiation of human mesenchymal stem cells [44]. In another study, a hybrid composite material made of gelatin-based hydrogel and maleimide-coated Ag NPs was prepared. The presence of NPs had significantly enhanced the mechanical property of the hydrogel. At the same time, the leakage of NPs can be avoided due to the immobilization of NPs in the matrix of the hydrogel [45]. 

By combining the hydrogel and NPs, the property enhancement of the materials can be achieved. At the same time, the limitations of the hydrogel scaffold, such as poor mechanical strength and lack of bioactivity, can be overcome [41]. As noble metal NPs were shown to have potential in tissue engineering applications as has been discussed earlier, it is also worthy to investigate whether its composite hydrogel can be used for tissue regeneration, and this will be discussed in following sections.

## 3. Synthesis Methods of Noble Metal NPs–Hydrogels Composites

**Figure 1:**
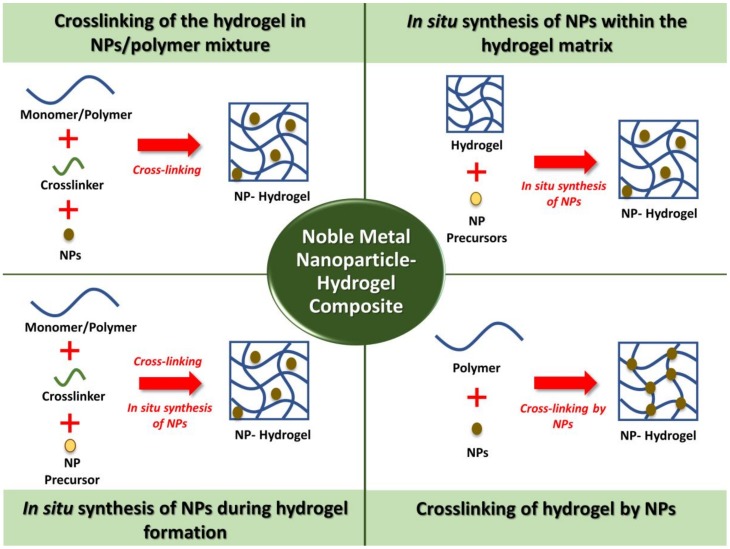
The noble metal nanoparticles (NPs) can be incorporated into the crosslinked hydrogel matrix using different approaches.

There are a few main approaches that have been adopted for the preparation of NP–hydrogel composites. In this section, common examples of the preparation methods for biomedical applications are discussed and summarized in Figure 1.

### 3.1. Crosslinking of the Hydrogel in NPs/Polymer Mixture

One of the simplest methods to fabricate the NP–hydrogel composite is the crosslinking of the polymer solutions containing preformed metal NPs. For example, Souza et al. have developed polyvinyl alcohol (PVA)/gellan gum hydrogel-containing Au NPs for drug delivery. Au NPs were mixed with PVA and gellan gum before hydrogel crosslinking [46]. Besides, chitosan was added to the suspension of Ag inlaid with Au NPs, before the mixture was brought to the freeze-drying process [47]. This approach is also commonly used for the hydrogel that undergoes a phase transition with temperature changes outside of a specific range, namely thermosensitive hydrogel [48]. This is shown in the study that involved the addition of Au NPs into methylcellulose solution [49]. The homogenized solution was brought to 37 °C from 4 °C for the gelling process to occur. Similarly, Arafa et al. used this approach to prepare Au NP-loaded thermoresponsive gels consisting of Pluronic®127 and hydroxypropyl methylcellulose for wound-healing transdermal drug delivery [48]. However, the drawback of this method is possible aggregation of NPs before or during the gelation process [50]. The NPs might also sediment due to the gravitational effect. Hence, NPs with a size within 100 nm should be used to achieve a stable dispersion [51]. In addition, for hydrogel with low crosslink density, NPs might easily leach out from the hydrogel [50]. 

### 3.2. In Situ Synthesis of NPs within the Hydrogel Matrix

A NP–hydrogel composite can also be easily fabricated by a two-step method. Firstly, the NP precursor solution was loaded into the crosslinked hydrogel. Then, an in situ reduction process of the metal ions occurred, resulting in the formation of NPs throughout the hydrogel matrix. For example, Varaprasad et al. developed a poly(acrylamide)/poly(vinyl sulfonic acid sodium salt) (PAAm-PVSA) hydrogel–Ag NP–curcumin composite for anti-bacterial wound dressing purposes. A swollen hydrogel was firstly prepared, followed by the soaking of the hydrogel in silver nitrate solution and sodium borohydride sequentially [52]. In another similar study, a sulfuric acid crosslinked chitosan hydrogel has also been soaked in silver nitrate solution until it reached swelling equilibrium. However, the reducing agent trisodium citrate has been used instead [53]. A greener approach has also been adopted by Bajpai and Kumari by using clove extract as the natural reducing agent [54]. Typically, the composite hydrogel will turn from colorless to yellowish-brown upon the formation of Ag NPs [55]. In this approach, the problem of NPs aggregation could be avoided, as the free space within the hydrogel porous structure offers a nanoscopic pot for the synthesis of NPs [50].

### 3.3. In Situ Synthesis of NPs during Hydrogel Formation 

The synthesis of NPs can also occur during the crosslinking process for hydrogel formation. This method is cost-effective and quick, as the composite hydrogel can be fabricated in a single-pot process. For instance, Ag NPs was synthesized during the formation hydrogel of carboxymethylcellulose (CMC) with phthalated-cashew gum. CMC, glycerine, cashew gum, and silver nitrate were mixed before the addition of sodium borohydride [56]. Besides, Dai et al. adopted this approach to fabricate a guar gum/Ag NPs hydrogel. Sodium borohydride not only behaved as the reductant for the synthesis of Ag NPs, it also contributed to the crosslinking of the hydrogel, because the sodium metaborate that was formed from sodium borohydride has acted as the crosslinker of guar gum molecular chains as well [57]. On the other hand, an Ag NP-loaded CMC hydrogel was fabricated by heating the mixture of CMC, propylene glycol, silver nitrate, and water. In this case, CMC acted as the gelling agent and reducing agent for silver nitrate. Therefore, the use of toxic reducing agents for NP synthesis can be avoided [58]. 

The irradiation method has been discovered as an alternative to the chemical reducing agent. Khampieng et al. have demonstrated the synthesis of Ag NP-embedded poly(vinyl pyrrolidone) (PVP) hydrogel dressing using gamma irradiation. PVP solution was mixed with silver nitrate and subjected to irradiation. The formation of Ag NPs and crosslinking of hydrogel have occurred simultaneously [59]. In another work, carboxymethyl sago pulp solution was mixed with silver nitrate solution and irradiated with electron beam radiation for the reduction process to occur to form Ag NPs [60]. Using a similar approach, Kumaraswamy et al. have synthesized Au NP/PVA hydrogel nanocomposites using gamma irradiation. This has been explained by the formation of free radicals due to the interaction of gamma irradiation with water [61]. The free radicals have recombined and formed reductive radicals that strongly induce the reduction of metal ions to metal NPs. As results, the NPs were immobilized within the hydrogel matrix [62]. This method is advantageous because the process is simple, environmental-friendly, and toxic initiator and crosslinking agents are not needed [59].

### 3.4. Crosslinking of Hydrogels by NPs

Another interesting method that has been applied for the preparation of NP–hydrogel composites is to use the metal NPs as the crosslinker. For example, Skardal et al. have utilized the multivalency of Au NPs and applied them to crosslink a printable semi-synthetic extracellular matrix hydrogel consisted of thiol-modified biomacromonomers derived from hyaluronic acid and gelatin for tissue engineering application [63]. Xing et al. have synthesized self-assembling collagen–Au hybrid hydrogel with tunable mechanical properties. This was achieved by the electrostatic interaction between positively charged collagen chains and [AuCl_4_]^−^ ions, followed by the reduction of [AuCl_4_]^−^ ions by the collagen hydroxyproline residues to form Au NPs that act as the crosslinkers of collagen chains [64]. 

Recently, this approach has been adopted to crosslink deoxyribonucleic acid (DNA)-Au NP hydrogel. This created new potential applications of this composite material such as bioimaging, diagnostics, and therapeutics [65]. Other than using bare metal NPs, some studies have utilized the functionalized NPs for crosslinking reaction. For example, Au NPs have been functionalized with carboxylic groups using mercaptoundecanoic acid, which is a thiol derivative. An esterification process has occurred between the carboxylic groups on Au NPs and hydroxyl groups of PVA that resulted in composite hydrogel [66]. Other than that, tiopronin-protected (N-(2-mercaptopropionyl)glycine) Au NPs was added to collagen type I solution and the crosslinking reaction has occurred via 1-ethyl-3-(3-dimethyl aminopropyl) carbodiimide (EDC) coupling [67]. This approach mainly depends on the ability of the NPs to connect the polymer chains and be adsorbed on the polymers [41].

## 4. Application of Noble Metal NP–Hydrogel Composites in Tissue Engineering 

The potential of noble metal NPs and hydrogel in tissue regeneration have stimulated the high interest of the researchers to further characterize the property of the hybrid of these materials. To address this, the studies on NP–hydrogel composites for regeneration of tissues such as soft tissues, bone tissues, and cardiac tissues are discussed as below.

### 4.1. Soft Tissues

Biocompatibility is an important criterion for the design of biomaterials for soft tissue transplantation. Xu et al. have tested the cytocompatibility of Ag NPs loaded poly(hydroxyethyl methacrylate) hydrogel with mouse embryo fibroblasts. The strong anti-bacterial response toward *Escherichia coli* and *Staphylococcus aureus* was also reported. The in vivo results have shown that the composite material was efficient at resisting foreign-body reactions and the formation of collagen capsule, allowed cell migration and infiltration [68]. Recently, a thermosensitive chitosan/phosphate hydrogel composites containing Ag and Ag–palladium core–shell NPs were tested with skin fibroblasts, hepatocellular carcinoma, and breast cancer cell lines that exhibited excellent cell viabilities [69]. For the study conducted by Zulkifli et al., the synthesized antimicrobial hydroxyethyl cellulose–Ag NPs scaffold has promoted the growth and proliferation of human fibroblasts. It was suggested that the surface roughness of scaffolds due to the presence of Ag NPs has contributed to the enhanced cell adhesion and proliferation [70]. As demonstrated in the study conducted by Alarcon et al., the anti-bacterial collagen-coated Ag NPs containing collagen hydrogel with Ag concentration <0.4 µM exhibited biocompatibility with primary human epidermal keratinocytes and dermal fibroblasts. In addition, the hydrogel containing 0.2 µM of Ag NPs had similar mechanical property as human skin, and it exerted an anti-inflammatory effect in vivo, as evidenced by the reduced level of pro-inflammatory cytokine IL-6 and other inflammation markers [71]. Based on the positive effect of the Ag NP hydrogels on fibroblasts proliferation, they could find potential in skin regeneration. The nanocomposite hydrogels containing Ag NPs have also been studied for their application in soft tissue engineering. Kumar et al. have synthesized the agarose hydrogel embedded with chitosan-coated Ag NPs. The mechanical strength of the scaffold fell within the range of native soft tissues, and it has provided sustained cell growth of HeLa, MiaPaCa2, and HEK cells. In addition, broad-spectrum anti-bacterial activity and high hemocompatibility were reported [72]. 

On the other hand, in a long-term in vivo study conducted by Grant et al., the Au NP-containing hydrogel was injected into the swine ears. The presence of Au NPs in the construct has probably improved the longevity of the material, since Au NPs have hindered the binding sites of collagenase. Interestingly, the irritation level of the material was retained at the low level, suggesting the nanocomposite as a potential biocompatible soft tissue filler [73]. In a study for angiogenesis, peptide sequence Arg-Glu-Asp-Val (REDV) was conjugated onto Au NPs to form a multivalent ligand, and this was used to construct the multivalent ligand-modified alginate hydrogel. The composite material has resulted in selective adhesion and enhanced the proliferation of human umbilical vein endothelial cells. Besides, the Au NPs–alginate surface has been shown to have an improved cell adhesion rate and cell spreading as compared to the alginate surface alone, which may be due to the interaction between Au NPs with vascular endothelial growth factor receptors on the cells’ plasma membrane. Besides, this might also be caused by the increased stiffness of the scaffold by Au NPs [74]. 

Based on the gathered findings, the hydrogels containing Au and Ag NPs showed their potential in different types of soft tissue engineering due to their biocompatibility and multiple bioactivities such as antimicrobial and anti-inflammatory properties. 

### 4.2. Bone Tissues

The number of cases of bone fractures is increasing every year due to the high frequency of accidents and diseases. Until today, the repair of the infected bone defect remains challenging, creating a demand for the functional biomaterials with osteogenesis and anti-bacterial properties for infected bone repair [75]. Therefore, it is interesting to study the performance of the hydrogel scaffolds incorporated with Ag or Au NPs for applications in bone regeneration. 

González-Sánchez et al. have demonstrated the synthesis of Ag NPs-based methacrylate hydrogels as a potential biomaterial for bone graft applications. The composite hydrogel exhibited excellent biocompatibility with osteoblast cells. In addition, anti-bacterial activity was reported when the NPs were incorporated by the post-mineralization absorption method [76]. In another study, a silk fibroin/nanohydroxyapatite hydrogel was modified with the Ag and Au NPs forming in situ. Similarly, the nanocomposite hydrogel has shown enhanced mechanical stiffness due to the presence of NPs. The materials also allowed the attachment and spreading of osteoblast cells [16]. Recently, an Ag NPs loaded polydopamine-coated poly (ethylene glycol) diacrylate hydrogel was fabricated for maxillary bone repair. The nanocomposite hydrogel not only exhibited bacteriostatic effect, it also promoted the osteogenesis of osteoblast cells through the upregulation of the expression of osteogenic genes of bone sialoprotein gene, alkaline phosphatase, osteocalcin, and runt-related transcription factor 2 [75]. 

The positive effect of metal NP-containing scaffolds was not limited to the osteoblast cells. Tentor et al. have demonstrated the enhanced proliferation and growth of preosteoblastic mouse cells cultured on chitosan/pectin thermosensitive hydrogels containing Au NPs [77]. On the other hand, Srinivasan et al. have reported the enhanced attachment and proliferation of human primary osteoblasts and human periodontal ligament cells on α-chitin and β-chitin hydrogel/bioactive glass ceramic/Ag NP composites. The high attachment and spreading of the cells on the scaffolds might be due to the increase in roughness and surface area provided by bioactive glass ceramic and Ag NPs [78]. Recently, stem cells such as adipose tissue-derived stem cells and bone marrow-derived mesenchymal stem cells have been studied for bone tissue engineering due to their ability to differentiate into osteogenic lineages [79]. In a study conducted by Heo et al., the photocurable gelatin hydrogel was loaded with Au NPs. There was an enhanced proliferation, osteogenic differentiation, and alkaline phosphatase activities of human adipose-derived stem cells, as they differentiate toward osteoblast cells. The finding was further supported by their results of in vivo studies, as the total regenerated bone volume at the bone defect sites of New Zealand rabbits was significantly improved in an Au NPs dose-dependent pattern. It was highlighted that the effect of Au NPs was comparable to the osteoinductive protein BMP-2. Hence, it might able to act as the potential alternative for BMP-2 in bone tissue engineering [80]. In another study, Au NPs were modified with N-acetyl cysteine (NAC) and loaded into gelatin hydrogel. Similarly, the osteodifferentiation of human adipose-derived stem cells on the composite hydrogel was observed, showing that the bone differentiation-promoting effects of Au NPs were preserved even when loaded into the hydrogel [81]. The effect probably was due to the Au NPs, which have acted as the promoter of osteogenic differentiation of mesenchymal stem cells through the activation of the p38 MAPK pathway [23,82]. 

Taken all together, Au and Ag NPs were shown to be beneficial to bone tissue engineering, and they were able to retain their bioactivities after being encapsulated into the 3D structure of the hydrogel.

### 4.3. Cardiac Tissues

In the human body, the heart is a strong power pump due to the myocardium that is made up of tightly packed uniaxial cytoarchitecture and electrically conductive Purkinje fibers, which supply an electrical conductive signal through the whole heart [83]. The regeneration of damaged cardiac tissue after myocardial infarction remains challenging due to the lack of electrical property of most of the implanted scaffolds. Hence, conductive materials have been added to the scaffolds to improve their electrical properties [84]. As Ag and Au are conductive, the application of an Ag and Au NP–hydrogel composite in cardiac tissue engineering has also been explored [85]. For example, You et al. have developed an electroactive Au NP impregnated thiol 2-hydroxyethyl methacrylate (HEMA)/HEMA composite hydrogel with tunable conductive and mechanical properties. The neonatal rat cardiomyocytes cultured on this conductive scaffold have shown an enhanced expression of connexin-43 without any electrical stimulation [86]. Shevach et al. have deposited Au NPs on the decellularized matrix to form a hybrid cardiac patch for myocardial infarction. The neonatal rat cardiomyocytes cultured within the scaffold showed elongated morphology, with organized connexin-43. This composite scaffold also has a stronger contraction force, lower excitation threshold, and faster calcium transients compared to pristine patches [84]. 

Recently, a study has been conducted on the collagen hydrogel composites containing peptide-modified nanoAu and nanoAg. The neonatal rat cardiomyocytes seeded on the scaffold showed enhanced proliferation, and increased the level of connexin-43 in the presence of electrical stimulation [87]. These findings are supported by the studies conducted by Navaei et al. The seeding of neonatal rat cardiomyocytes on the Au nanorod-incorporated gelatin methacrylate hydrogels has resulted in the formation of uniform, dense, and aligned cardiac tissues, with a homogenous distribution of arcomeric α-actinin and connexin 43, which exhibited enhanced cytoskeletal alignment and cellular connectivity. In addition, the nanocomposites have supported the synchronous tissue-level beating of cardiomyocytes [88,89]. The composite material has also been fabricated using a bioprinting method, in order to create a 3D-printed functional cardiac tissue construct made of Au nanorod-incorporated gelatin methacryloyl (GelMA)-based bioink. In the Au nanorod-containing printed construct, the cardiac cells exhibited enhanced cell adhesion and organization compared to the construct without an Au nanorod. In addition, there was a higher expression level of connexin 43 and higher synchronized contractile frequency compared to the pristine GelMA/alginate bioink-printed constructs. The reported contractile forces might be attributed to the inhibition of excessive cardiac fibroblasts by Au nanorods [90]. 

Other than cardiomyocytes, the effect of Au nanocomposites on stem cells has also been explored. A thermoresponsive hydrogel composite made of chitosan and chitosan-stabilized Au NPs has been synthesized and characterized. The incorporation of Au NPs has promoted the differentiation of mesenchymal stem cells into cardiac lineages. This effect could also be due to the presence of electrical cues in the matrix as contributed by the electroconductive property of the Au NPs [91]. Based on the reported studies, the conductive NP–hydrogel composites mainly play their role to enhance the expression of connexin 43 due to the conductivity present within the hydrogel matrix. This creates a new therapeutic opportunity in cardiac tissue engineering. 

**Figure 2:**
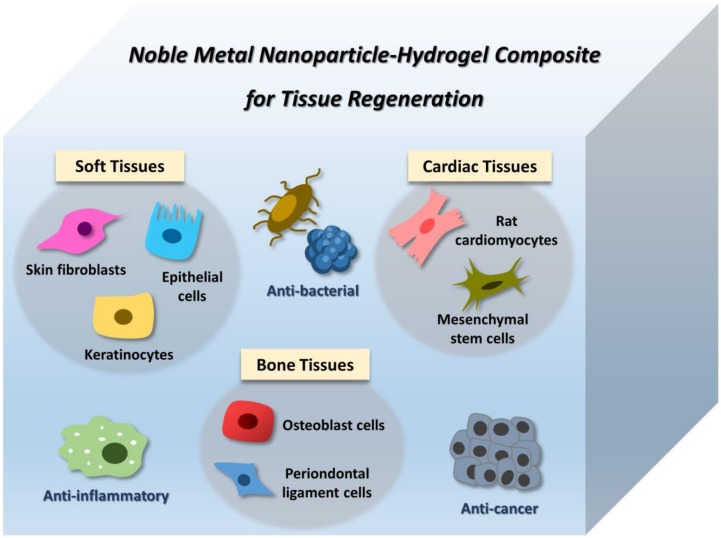
Noble metal nanoparticle (NP)–hydrogel composites for tissue regeneration. The composites were shown to have potential for the regeneration of tissues such as soft tissues, bone tissues, and cardiac tissues. At the same time, the composites also offer some other exciting bioactivities such as anti-bacterial, anti-inflammatory, and anti-cancer properties.

The potential of Au and Ag-containing hydrogels in applications such as skin, bone, and cardiac regeneration have been particularly highlighted and summarized in Table 1 and Figure 2.

## 5. Limitations and Challenges

Based on the studies as discussed, the incorporation of noble metal NPs such as Ag and Au NPs have added advantageous functionality to the hydrogel scaffolds. However, there are concerns on the cytotoxic effect of the metal NPs, because the interaction of NPs with cells remains controversial, and the mechanism has not yet been fully understood [46,92]. Indeed, the discrepancy of the results could be due to the variation in parameters such as size, shape, and surface charge [93]. In addition, different types of cells display distinct cytotoxic response. For example, Vero cells, which are a type of kidney epithelial cells from the African green monkey, were shown to be more susceptible to the biocompatible chitosan/pectin/Au NPs hydrogel than LLCMK_2_ cells, which are the kidney epithelial cells from *Macaca mulatta* [77]. Other than that, the Ag-containing hydrogel wound dressing was found to exert a different level of toxicity toward immortal keratinocytes and primary keratinocytes. Therefore, it is crucial to select an appropriate model cell line for more accurate results of biocompatibility tests [94]. This cellular cytotoxicity study is essential before advancing to using in vivo models. 

In NP–hydrogel composites, the NPs were entrapped in the 3D structure of the hydrogel, and massive NPs uptake by cells could be avoided upon implantation [92]. However, the uncontrolled release of NPs from the scaffold could also be another concern. The NPs might not only directly be taken up by the cells in exposed organs, but also translocated to other organs, causing undesired toxicity or other adverse effects [95]. Kostic et al. have conducted a study to assess the translocation of Ag from hydrogel composite, and it has been concluded that the release of Ag was dependent on the hydrodynamic conditions at the implantation site [96]. In the in vivo study conducted by Alarcon et al., the systemic distribution of Ag NPs after the implantation of an Ag NP–collagen hydrogel was investigated. Ag was found mainly accumulated within the tissues around the implant. Besides, Ag was also detected in the liver, kidney, and spleen within 24 h [71]. This indicated that the safety assessment of the scaffold has to be extensively conducted on the affected tissues or organs, and not limited only to the tissue regeneration site.

## 6. Conclusions

As evident in the research studies presented in this review, the incorporation of noble metal NPs into hydrogel formulations appeared to be promising in enhancing the functionality of the materials from physical and biological aspects. However, most of the studies presented only involved short-term cell-based and in vivo studies. There is still a large gap between the knowledge of the short-term and long-term effect of the NP–hydrogel composites. Besides, to date, knowledge on the interaction of the materials with cells has been only limited to a few commonly studied cell types. Hence, further studies are crucial in order to fill in this gap, as the complex process of tissue regeneration in real life takes more time and involves the interaction of multiple cell types. 

## Figures and Tables

**Table 1:** Summary of the research studies on NP–hydrogel composites for tissue engineering applications.

Tissue Regeneration	Nanoparticles	Scaffolds	Synthesis Method	Cell Line/Animal Tested	Effect of NPs Addition on the Physical Property of Material	Effect of NPs Addition on the Biological Property of Material	Reference
**Soft Tissues**	Collagen-coated Ag NPs	Collagen	Crosslinking of the hydrogel in NPs/polymer mixture	Primary human epidermal keratinocytes; Dermal fibroblasts; Mice	Hydrogel containing 0.2 µM Ag NPs has similar Young’s modulus as human skin	Biocompatibility, anti-inflammatory, and anti-bacterial activities	[71]
	Ag NPs	Poly(hydroxyethyl methacrylate)	In situ synthesis of NPs during hydrogel formation	Mouse embryo fibroblasts (NIH-3T3); BALB/c female mice	Increased amounts of Ag NPs loading slightly enhanced the compressive modulus of hydrogel	Biocompatibility, anti-bacterial, and in vivo resistance to foreign-body reactions	[68]
	Ag NPs	Hydroxyethyl cellulose	Crosslinking of the hydrogel in NPs/polymer mixture	Human fibroblasts	Glass transition temperature of scaffold increases as concentration of AgNO_3_ increases	Biocompatibility	[70]
	Ag NPs & Ag-Palladium NPs	Chitosan/Hydroxyapatite & Chitosan/Beta-tricalcium phosphate	Crosslinking of hydrogel in NPs/polymer mixture	Normal skin fibroblasts (BJ1); Hepatocellular carcinoma cells (HEPG2); Breast cancer cells (MCF7);	N/A	Biocompatibility and anti-bacterial activity	[69]
	Chitosan-coated Ag NPs	Agarose	Crosslinking of the hydrogel in NPs/polymer mixture	Human cervical carcinoma cells (HeLa); Human pancreatic epithelial carcinoma cells (MiaPaCa2); Human embryonic kidney cells (HEK);	Mechanical strength (five to eight Mpa) falls within range for soft tissue engineering	Biocompatibility, anti-bacterial activity, and hemocompatibility	[72]
	Au NPs	Alginate	Crosslinking of the hydrogel in NPs/polymer mixture	Human umbilical vein endothelial cells (HUVECs)	N/A	Enhanced HUVECs adhesion rate and cell spreading	[74]
	Au NPs	Collagen	Conjugation of Au NPs to collagen fibrils	Swine	Enhanced longevity of the material	Biocompatibility and low irritation	[73]
**Bone Tissues**	Ag NPs	α-chitin and β-chitin/Bioactive glass ceramic NPs	Crosslinking of hydrogel in NPs/polymer mixture	Human periodontal ligament cells (hPDL); Human primary osteoblasts (POB)	Composite scaffold has decreased porosity and enhanced compressive strength.	Anti-bacterial activity, differentiation, and mineralization of POB in the absence of osteogenic supplements	[78]
	Ag NPs	Poly (ethylene glycol)	In situ synthesis of NPs within the hydrogel matrix	Osteoblast cells (MC3T3-E1); Sprague–Dawley rats	N/A	Anti-bacterial activity, promoted osteogenesis in vitro and in vivo	[75]
	Ag NPs	Methacrylate	Crosslinking of hydrogel in NPs/polymer mixture; diffusion reaction; adsorption of NPs	Osteoblast cells (MC-3T3)	No effect on mechanical properties (absorption method)	Biocompatibility and anti-bacterial activity (absorption method)	[76]
	Au NPs	Chitosan/Pectin	Crosslinking of the hydrogel in NPs/polymer mixture; diffusion reaction; adsorption of NPs	Normal kidney epithelial cells (VERO); Epithelial colorectal adenocarcinoma cells (HT-29); HPV-16 positive human cervical tumor cells (SiHa); Kidney epithelial cells (LLCMK2); Murine macrophage cells (J774A1 cells); Mouse preosteoblastic cells (MC3T3-E1)	Gelation temperature decreases with decrease in pectin concentration and increase in Au NPs levels	Biocompatibility and promoted growth of MC3T3-E1 cells	[77]
	Au NPs	Gelatin	Crosslinking of the hydrogel in NPs/polymer mixture	Human adipose-derived stem cells (ADSCs); New Zealand Rabbit	N/A	Biocompatibility, promoted differentiation toward osteoblast cells, and improved bone regeneration in vivo	[80]
	N-acetyl cysteine-Au NPs	Gelatin-tyramine	Crosslinking of hydrogel in NPs/polymer mixture	Human adipose derived-stem cells (hASCs)	N/A	Biocompatibility and promoted osteodifferentiation	[81]
	Ag and Au NPs	Silk fibroin/Nanohydroxyapatite	In situ synthesis of NPs within the hydrogel matrix	Osteoblast-like cells (MG63)	Hydrogels containing Ag and Au NPs have enhanced mechanical stiffness	Biocompatibility and anti-bacterial activity	[16]
**Cardiac Tissues**	Peptide-modified Ag and Au NPs	Collagen	Crosslinking of the hydrogel in NPs/polymer mixture	Neonatal rat ventricular cardiomyocytes and cardiac fibroblasts	Enhanced mechanical and electrical properties of the material	Promoted reparative macrophage migration	[87]
	Au NPs	Decellularized omental matrices	Evaporation of Au for deposition	Neonatal rat ventricular cardiomyocytes, Cardiac fibroblasts	Au NPs patches have enhanced conductivity and similar longitudinal elastic modulus as pristine patches	Aligned cardiac cells with organized connexin 43 and attenuation of fibroblast proliferation	[84]
	Au NPs	Thiol 2-hydroxyethyl methacrylate (HEMA)/HEMA	In situ synthesis of NPs within the hydrogel matrix	Neonatal rat ventricular cardiomyocytes	Conductive hydrogel has tunable conductive and mechanical property, with Young’s modulus similar to myocardium	Increased expression of connexin 43	[86]
	Chitosan-modified Au NPs	Chitosan	Crosslinking of the hydrogel in NPs/polymer mixture	Mesenchymal stem cells	Tunable electrical conductivity of the hydrogel by different concentration of Au NPs	Biocompatibility, enhanced differentiation into cardiac lineages	[91]
	Au nanorods	Gelatin methacrylate	Crosslinking of the hydrogel in NPs/polymer mixture	Neonatal rat ventricular cardiomyocytes	Enhanced mechanical and electrical properties of the material	Enhanced formation of cardiac tissues	[88,89]
	Au nanorods	Gelatin methacryloyl	Crosslinking of the hydrogel in NPs/polymer mixture (3D bioprinting)	Neonatal rat ventricular cardiomyocytes and cardiac fibroblasts	Nanocomposite bioink has increased shear-thinning effect and enhanced printability	Enhanced cell adhesion and organization, electrical propagation, and synchronized contraction	[90]
